# The emerging treatment landscape of targeted therapy in non-small-cell lung cancer

**DOI:** 10.1038/s41392-019-0099-9

**Published:** 2019-12-17

**Authors:** Min Yuan, Li-Li Huang, Jian-Hua Chen, Jie Wu, Qing Xu

**Affiliations:** 1 Department of Oncology, Dermatology Hospital, Tongji University School of Medicine, Shanghai, 200443 China; 2 Department of Oncology, Shanghai Tenth People’s Hospital, Tongji University School of Medicine, Shanghai, 200072 China; 3 Peggy and Charles Stephenson Cancer Center, University of Oklahoma Health Sciences Center, Oklahoma City, OK USA; 4 Department of Pathology, University of Oklahoma Health Sciences Center, Oklahoma City, OK USA

**Keywords:** Lung cancer, Target identification

## Abstract

Lung cancer is one of the most common cancer in the world. In 2018, there were over 2 million new cases of lung cancer and over 1.7 million deaths were attributed to lung cancer. Targeted therapy has emerged as an important mean of the disease management for patients with non-small-cell lung cancer (NSCLC). Herein, we review and analyze recent literature, discuss the targeting pathways and ongoing clinical trials in lung cancer. Chemotherapy is no longer the best available treatment for all patients. Therapeutic decisions should be guided by an understanding of the molecular features of patient’s tumor tissues. The future gains will likely emerge from finding optimal ways of combining targeted therapy, immunotherapy, and chemotherapy.

## Introduction

**Fig. 1:**
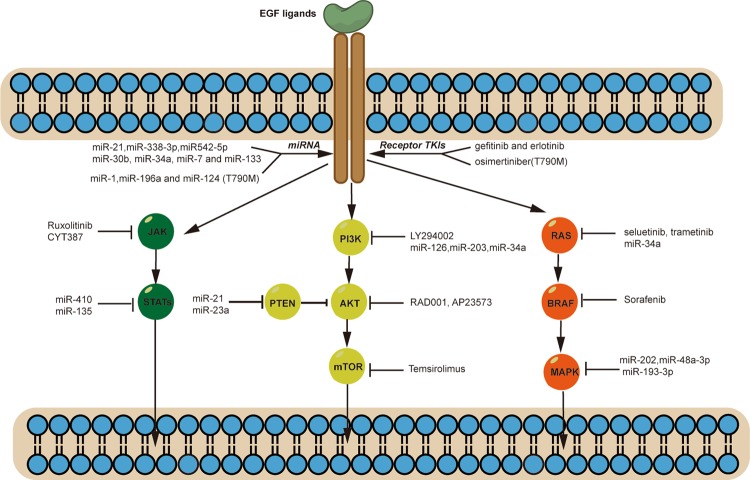
Therapies targeting the key oncogenic signaling pathways in lung cancer. There are several abnormal signaling and cell physiology-related pathways in lung cancer. Drugs targeting these abnormal pathways are depicted schematically. These drugs include agents specifically inhibiting components of the EGFR pathway and other family members and/or members of the VEGFR pathways. Other agents in development include inhibitors of the PI3K/AKT/mTOR pathway, the RAS/BRAF/MAPK pathway, and the JAK–STAT pathway.

Lung cancer is one of the most deadly and common types of cancer in the world.^1^ In 2018, there are over 1.7 million people died from lung cancer.^2^ Based on cell origin, about 80–85% are of non-small-cell lung cancer (NSCLC).^3^ NSCLC is further divided into lung adenocarcinomas, squamous cell carcinoma and large cell carcinoma based on their histological features.^4^ With the advent of genomic medicine, precisionlized oncology has helped improve treatment outcomes and quality of life compared to traditional chemotherapy.^5^ Advances in the knowledge of pathways, technologies for detecting actionable genetic lesions, and newly developed drugs to block the activities of the pathways in recent years have allowed the physicians to tailor the treatment options.^6^ In lung adenocarcinoma, a number of targetable major pathways have been identified, such as EGFR, PI3K/AKT/mTOR, RAS–MAPK, and NTRK/ROS1 pathways.^7–10^ Many drugs targeting these pathways have been developed and shown clinical benefits.^11^ Some of them have now replaced chemotherapy as the first line treatment, such as EGFR inhibitors erlotinib, gefitinib, PI3K/AKT/mTOR inhibitors everolimus, and NTRK/ROS1 inhibitors entrectinib.^17–20^ Nevertheless, while target therapy in NSCLC has provided disease control, the tumors inevitably develop drug resistance. Understanding resistance mechanisms and developing combinational therapies are essential for improving the treatment outcomes.^16^ Mechanisms of drug resistance in NSCLC have been identified such as TK domain mutation (T790M), MET amplification, RAS mutation.^17–20^ Other target therapy drugs are in clinical development and have shown promising clinical results to drug resistance, such as third-generation EGFR-TKIs (Osimertinib) which could active and target both EGFR sensitive and T790M resistant mutation.^21^ With the emergence of immunological checkpoint inhibitors, many NSCLC patients are responsive to antibodies such as the anti-PD1 antibodies nivolumab and pembrolizumab.^22^ In addition, some studies have reported that some targeted therapies with immunotherapies are efficacious in NSCLC.^23^ Therefore, this review will focus on the gene mutations in important pathways in NSCLC, and discussed emerging therapies for these tumors (Fig. 1).

## Targeting pathways in non-small-cell lung cancer

### EGFR pathways

EGFR is a member of tyrosine kinase type I receptors family, and its gene is located on the short arm of human chromosome 7.^24^ In EGFR, there are 28 exons that form a protein that is distributed on the cell membrane of various epithelial cells, where it binds to epidermal growth factor or heparin-binding EGF and regulates the growth of cells.^25^ By comparison, exon 20 insertions and exon 18-point mutations are less common than exon 19 deletions and exon 21 L858R substitutions in terms of EGFR mutations in NSCLC.^26,27^ Activation and regulation of EGFR and downstream genes lead to cell proliferation, apoptosis, and angiogenesis.^28^ Some measures have been developed to target EGFR, such as tyrosine kinase inhibitors (TKIs), BRAF inhibitors.^29,30^

In past decades, tyrosine kinase inhibitors have been considered efficient drugs in NSCLC and have served as excellent targeted drugs.^31^ Various agents targeting EGFR have emerged out such as gefitinib, erlotinib, cetuximab and panitumumab.^32–34^ Some studies demonstrated that the two first-generation EGFR-TKIs (gefitinib and erlotinib) had substantial benefits in terms of PFS compared to chemotherapy as first-line therapy.^35^ Unfortunately, the OS in advanced NSCLC patients was not obviously affected by EGFR-TKI treatment after chemotherapy.^36^ Some studies have shown that patients develop drug resistance after receiving first-generation EGFR-TKI therapy for 10–14 months.^37,38^ Mechanisms of drug resistance of first-generation EGFR-TKI in NSCLC have been identified such as TK domain mutation (T790M), MET amplification, RAS mutation.^39–41^ TK domain mutation (T790M) is described as the most common acquired resistance mutation in the NSCLC patients.^42,43^ A subset of NSCLC patients with the T790M mutation have never underwent EGFR-TKI treatment.^44,45^ These finding suggest that the T790M mutation is a potential target in NSCLC patients.^44^ Therefore, new measures and therapies need to develop to overcome drug resistance.

In recent years, osimertinib has emerged as a third-generation EGFR-TKIs that can active and target both sensitive and resistant (T790M) EGFR mutations.^46^ In the FLAURA study, the median PFS of NSCLC patients treated with osimertinib (18.9 months) was obviously longer than that in patients treated with first-generation EGFR-TKIs (gefitinib and erlotinib) (10.2 months).^47^ This group of patients had EGFR exon 19 deletions or L858R mutations in untreated NSCLC that had progressed. Another study (AURA3) showed that the PFS associated with osimertinib was 10.1 months, whereas the PFS associated with pemetrexed/platinum doublet chemotherapy was 4.4 months. The objective response was significantly higher in osimertinib group.^23^ Though osimertinib has showed promising survival benefits in clinical trials, choice still needs to be made. It is beneficial to consider the optimal sequence of EGFR TKIs in order to maximize their clinical benefit. Osimertinib followed by a first-or-second-generation EGFR-TKI or followed by pemetrexed/platinum doublet chemotherapy might be a rational choice.^48–50^ There remain debates about sequence of therapy, more clinical trials are required to clarify the most appropriate sequential therapy in patients with EGFR mutation-positive NSCLC.

Unfortunately, a study showed that drug resistance to osimertinib has emerged. Among 45 patients, the EGFR C797S mutation, mutations in PIK3CA, KRAS, BRAF, and MET amplification were found.^51^ In EGFR-mutated NSCLC, 5–20% EGFR-TKI drug-resistant patients developed MET amplification.^52^ MET amplification increases the proliferation and migration of HCC827 cell line in NSCLC.^53^ Results showed that aiming at MET amplification could prompt therapeutic efficacy.^54^ A MET inhibitor (crizotinib or SGX532) could increase the gefitinib sensitive in NSCLC.^55^ In addition, KRAS, a member of RAS mutation has been identified as the most frequently mutated oncogene in NSCLC.^56^ Previous studies have shown that KRAS mutation has been identified as a mechanism of EGFR-TKI resistance in NSCLC.^57^ Thus, therapeutic strategies target to KRAS mutation might be overcome drug resistance and improve the therapeutic efficacy.^58^ Some KRAS inhibitors have been identified, such as KRAS G12C mutant protein which showed excellent efficacy in vitro.^59^ Therefore, different therapeutic strategies targeting at EGFR pathway showed various efficacy which overcomes drug resistance and cancer progression.

### PI3K/AKT/mTOR pathways

The PI3K/AKT/mTOR pathway is considered a potential target in NSCLC. The PI3K signaling pathway regulates various cellular processes, including cell proliferation, differentiation, and apoptosis, as well as gene transcription and protein synthesis.^8,9^ The PI3K/AKT/mTOR pathways has been demonstrated to activate upstream receptors (EGFR and PDGFR) and to be mutated in several cancers, including breast cancer, gastric cancer and NSCLC.^10^ Studies have shown that PIK3CA mutations occur in approximately 4% of NSCLC tumors expressing PTEN protein, which inhibits the PI3K/AKT/mTOR pathways.^11,12^ Several measures have attempted to target the PI3K/AKT/mTOR pathways in NSCLC. A PI3K inhibitor (LY294002) has been reported to enhance the sensitivity of NSCLC to chemotherapy and radiation.^13^ In addition, another mTOR inhibitor, temsirolimus (CCI-779), which targets molecules downstream of the PI3K pathways, has shown promising antitumor activity in phase I trials of NSCLC.^14^ Other PI3K/AKT/mTOR inhibitors, everolimus (RAD001 or AP23573), is also in preclinical trails. Studies have shown that downregulation of miR-93 in NSCLC inhibits cell proliferation and apoptosis.^15^ miRNA-223 has been shown to suppress cancer cells by targeting the EGFR/AKT2 pathway in NSCLC.^16^ Some studies have demonstrated that the PI3K/AKT/mTOR pathway is an important player in EGFR-TKI resistance.^17,18^ Loss of the PTEN gene, the protein product of which functions downstream of the PI3K/AKT/mTOR pathway, has been shown to play an important role in erlotinib and gefitinib resistance in EGFR-mutated NSCLC.^19^

Several miRNAs have shown potential to reverse the PI3K/AKT/mTOR pathway-induced inhibition of tumor growth, progression, and metastasis. Moreover, miRNAs can regulate resistance in NSCLC by targeting the PI3K/AKT/mTOR pathway. Wang C and colleagues demonstrated that miRNA‑328 overexpression conferred cisplatin resistance to A549 cells via PTEN.^20^ Interestingly, an miRNA‑328 inhibitor upregulated the expression of PTEN and restored the sensitivity to cisplatin in NSCLC cells.^20^ In addition, in Shen’s experiment, miR-21 was shown to have an important role in inducing gefitinib resistance in NSCLC.^21^ Knockdown of miR-21 by inhibiting the PTEN/PI3K/AKT pathways increased the sensitivity to gefitinib in vitro and in vivo.^21^ Han and colleagues demonstrated that miR-23a served as an inhibitor of PTEN in the PC9 NSCLC cell line.^22^ Downregulation of miR-23a reversed the resistance of erlotinib. Thus, miR-23a could be a potential target for overcoming resistance to EGFR-TKIs in NSCLC.^22^ In addition, there are several miRNAs that target the PI3K/AKT/mTOR pathways that have shown promising antitumor efficacy and the ability to reverse EGFR-TKIs resistance. Knockdown of miR-126, miR-203, and miR-34a has been demonstrated to regulate drug resistance via PI3K/AKT signaling.^23–25^ Furthermore, miRNAs can regulate upstream factors of the PI3K/AKT/mTOR pathways in NSCLC. The C-MET gene is known to be involved in resistance to EGFR-TKIs, and it was targetable by miR-34a in HCC827 and PC-9 cells. miR-34a successfully inhibited the EGFR/PI3K/AKT pathway and reversed gefitinib resistance in NSCLC.^26^

### RAS–MAPK pathways

The human RAS gene family, including KRAS, NRAS, and HRAS, encodes small plasma membrane G proteins (GTPases) that regulate cell proliferation and progression through different pathways^27^. Some upstream membrane receptors are associated with the RAS family, for example, EGFR and FGFR. In addition, some downstream pathways are involved (the RAS–MAPK and the PI3K–AKT–mTOR pathways) in cell differentiation and survival via the RAS family.^79^ The most mutated RAS family gene in lung cancer is KRAS.^28^ KRAS mutations occur in 20–40% of lung adenocarcinomas, which make up ~20% of cases in Western countries and ~10% of cases in Asian countries.^80^ Some studies have showed that KRAS mutations may be factors in EGFR-TKIs resistance in targeted therapies in NSCLC.^29^ Therefore, various agents attempt to target the RAS pathway in NSCLC to overcome drug resistance. Many downstream receptors of the RAS pathways, including RAF, MEK, and mTOR, have been studied as potential targets in NSCLC in multiple clinical trials. Sorafenib is an oral drug that serves as a multikinase inhibitor by targeting RAF and other receptors including VEGFR-2, VEGFR-3, and PDGFR-b.^30^ In early clinical trials, sorafenib showed promising efficacy and was well tolerated, with good stability in patients.^82^ However, in the subsequent clinical studies of sorafenib, the results showed that there was no efficacy in NSCLC, with objective response rates (ORRs) less than 10%.^31,32^ In addition, other BRAF inhibitors have shown similar results in clinical trials, performing well in BRAF-mutant patients but not KRAS-mutant lung NSCLCs.^33^ For MEK inhibitors, the results of clinical studies of selumetinib showed no benefit over pemetrexed-based chemotherapy in untreated NSCLC. Similar results have been found with trametinib when the therapy was compared with docetaxel chemotherapy.^34^ This lack of efficacy could be because current Raf inhibitors only recognize one binding site and thus decrease binding affinity for other sites. Thus, there is an urgent need for a method or new target drug to solve these problems in RAS-mutated patients. Several genetic methods have been developed and could be used to discover potential targets in NSCLC. Paula T. Hammond and colleagues tried to combine siKRAS, miR-34a and cisplatin to target the KRAS/P53 mutation in NSCLC. Their results demonstrated that the combination of miRNA and chemotherapy enhanced the cancer cell line toxicity and increased the treatment efficacy, with a prolonged survival time in mice.^35^ Other studies showed that miR-202 enhanced the anti-tumor effect of cisplatin in NSCLC by targeting the Ras/MAPK pathway. In their study, the expression level of miR-202 was found to enhance the cisplatin sensitivity of NCI-H441 and A549 NSCLC cells. Furthermore, the overexpression of miR-202 was found to inhibit the Ras/MAPK pathway by targeting the KRas gene.^36^ A similar phenomenon has been observed with other miRNAs, including miRNA-48a-3p and miR-193a-3p.^37,38^ In Liang Ma’s study, the researchers found that miR-148a-3p inhibited the development and progression of NSCLC by reducing the expression of SOS (son of sevenless) via Ras/MAPK/Erk signaling.^90^ In addition, decreased expression of miR-193a-3p was found in the NSCLC tumor tissues and inhibited cell proliferation and progression. In addition, the expression of miR-193a-3p was found to be associated with KRAS mRNA, which indicated that miR-193a-3p could act as a tumor suppressor by targeting KRAS in NSCLC.^91^

### JAK–STAT pathways

Signal transducers and activators of transcription (STAT) proteins make up a family of 7 different proteins (STAT1, STAT2, STAT3, STAT4, STAT5A, STAT5B, STAT6, and STAT7) that recruit Janus kinases (JAKs) by activating different proteins.^39^ The JAK–STAT pathways is involved and activated in various solid tumors, including NSCLC.^40^ The JAK–STAT pathways plays important roles in cells differentiation, proliferation, and cancer progression. STAT3 is the most studied STAT family member and was found to play a role in malignancies of various types. In addition, in lung cancer, the STAT pathways regulates apoptotic genes, inhibits cell growth and improves the treatment efficacy of EGFR-TKIs.^41^ Reports have indicated that combining EGFR-TKIs or chemotherapy with JAK/STAT pathways inhibitors could enhance antitumor efficacy and decrease drug resistance in NSCLC.^42,43^ The role of JAK/STAT pathways inhibitors in cisplatin resistance was studied in NSCLC cell lines and tumor xenograft models. The authors demonstrated that a JAK2 inhibitor (ruxolitinib) blunted the growth of cisplatin-resistant H1299 cells and enhanced antitumor activity by inhibiting tumor growth and inducing caspase-3 expression in tumor models with cisplatin resistance.^44^ However, further study of the efficacy and safety of combination treatments with JAK2 inhibitors is needed. Furthermore, Yibang Chen and his collaborators investigated the antitumor efficacy of a combination of a JAK-2 inhibitor (CYT387) and cetuximab in NSCLC models both in intro and in vivo. The results demonstrated that cell toxicity increased when cetuximab was combined with CYT387 in resistant cell lines (H1975 and H1650). In addition, the antitumor activity was increased with the combination of a JAK/STAT pathway inhibitor with cetuximab in the resistant models. Thus, JAK–STAT pathway inhibitors could be a potential method in NSCLC therapy either alone or in combination with EGFR-TKIs.^45^ M. Li and his team studied the role of suppressor of cytokine signaling 3 (SOCS3) and miR-410 in regulating proliferation in NSCLC. SOCS3 negatively regulates the JAK–STAT pathways and was decreased in NSCLC tissue compared to normal tissue. They found that STAT3 phosphorylation was significantly reduced via treatment with anti-miR-410 and SOCS3 in lung cancer cells. These results indicate that miR-410 could serve a potential therapeutic target in NSCLC patients by regulating the JAK/STAT pathways. However, efficacy studies and clinical trials need to explore this potential in the future. Another study explored the potential role of miR-135 in NSCLC cells. The authors found that the expression of miR-135 was increased in NSCLC cells and that cell proliferation, invasion, and migration were suppressed with the silencing of miR-135. In addition, a subsequent study found that miR-135 inhibition upregulated TRIM16 expression through the JAK/STAT pathway and thus enhanced the sensitivity to gefitinib in NSCLC cell lines.^46^

Other measures attempt to enhance antitumor activity by combining a JAK/STAT inhibitor with gene therapy or immunotherapy. Manish R. Patel and his colleagues used a JAK/STAT inhibitor (ruxolitinib) in combination with vesicular stomatitis virus (VSV) to cure lung cancers. In their study, the results indicated that ruxolitinib increased the virotherapy efficacy in both resistant and sensitive NSCLC cells in vitro and in vivo. In addition, the combination therapy also enhanced PD-L1 expression and the levels of tumor immune infiltration in lung cancer.^47^ These results provide a method for NSCLC therapy, but further clinical evaluation of the combination of a JAK/STAT inhibitor with virotherapy or immunotherapy needs to be carried out.

### HER3

Over the last two decades, it has become increasingly evident that HER3 plays an important role in cancer biology. HER3 (also known as ERBB3) is a unique member of the HER family and is considered an inactive receptor.^48^ EGFR and HER4 both have several ligands, while HER3 has only one single ligand, called heregulin (HRG) or neuregulin (NRG). Receptor interaction can result in dimerization when a ligand binds to the extracellular region of EGFR or HER3/4.^49^ Dimerization is the first critical step for HER receptor activation of downstream signaling pathways, such as the JAK/STAT, MEK/MAPK, and PI3K/AKT pathways, and several others.^50^ To prevent dimerization, EGFR, HER3, and HER4 usually exist as molecularly folded monomers (inactive state).^51^ Unlike other HER family members (EGFR, HER2, and HER4), the HER3 receptor has little or no tyrosine kinase activity.^52^ Overexpression of HER3 can promote tumor progression by increasing metastatic potential in various human cancers and causing the failure of treatment. Studies have found highly activated HER3 with somatic mutations of EGFR in NSCLC.^53^ HER3 becomes potently trans-phosphorylated and activated by binding with mutated EGFR and mediates the PI3K/AKT pathway. Therefore, EGFR TKIs can achieve potent antitumor activity in EGFR-mutant NSCLC by preventing HER3/PI3K/AKT signaling activation.^54^ Even during ongoing EGFR-TKI therapy, HER3 can be activated by binding with an amplified MET oncogene, and this can perpetually activate the PI3K/AKT pathway.^55^ Thus, as a result of these characteristics of HER3 and its activity in cell survival signaling pathways, it might be a promising therapeutic target in EGFR-mutant NSCLC.

HER3 has been found to have an important role in resistance to EGFR-TKIs in NSCLC.^56,57^ Studies have revealed that an important mechanism resulting in resistance to EGFR-TKI therapy could be the compensatory upregulation of HER3 along with the sustained PI3K/AKT signaling.^58,59^ Because HER3 lacks kinase activity, targeting HER3 with a blocking antibody becomes a potential strategy being tested in current preclinical studies in cancer patients.^60–62^ Several phase I studies to evaluate monoclonal antibodies targeting HER3, such as patritumab, lumretuzumab, SAR256212 or LJM716, are ongoing. Results show acceptable safety of these agents either as monotherapies or in combination.^54,63–66^

U3-1287 (renamed as patritumab) was the first fully humanized anti-HER3 antibody. It has been examined in several clinical trials in patients with advanced solid tumors and is being currently studied in NSCLC.^61,67^ Patritumab has been shown in vitro and in vivo to overcome the HRG-dependent resistance to EGFR TKIs in NSCLC. These data further support the significance of ongoing clinical trials testing patritumab combined with EGFR-TKIs, such as erlotinib, to treat NSCLC patients with high expression of HRG.^54^ However, studies found that the antitumor activity of patritumab could be limited by combination treatment with erlotinib in patients with EGFR-TKI resistance acquired after therapy. A phase I clinical trial combining patritumab with erlotinib showed an overall response rate of 4.2% and a disease control rate of 62.5% in Japanese patients with advanced NSCLC.^68^ Similarly, a phase III study of patritumab combined with erlotinib in EGFR wild-type patients with locally advanced or metastatic NSCLC that had progressed on at least one prior systemic therapy was terminated.^69^ Thus, an alternative HER3-targeting therapy is required for EGFR-mutant NSCLC treatment.

U3-1402 is a new HER3-targeted antibody-drug conjugate with a fully human HER3-targeted antibody, a novel cleavable peptide-based linker, and a topoisomerase I inhibitor payload. There is an ongoing phase I trial of U3-1402 in patients with NSCLC.^70^ In a current study, it was reported for the first time that U3-1402 was an effective treatment for EGFR-mutant NSCLC.^71^ An ongoing multicenter phase I dose escalation and expansion study is assessing the safety/tolerability and preliminary activity of U3-1402 in metastatic or unresectable EGFR-mutant NSCLC patients who were T790M negative after disease progression while on erlotinib, gefitinib or afatinib; or who developed disease progression while on osimertinib regardless of T790M status.^72^ In 13 evaluable cases, all but one patient had a decrease in SLD, and two patients had confirmed the partial response. U3-1402 has shown preliminary antitumor activity and a manageable safety profile in EGFR TKI-resistant NSCLC.

## Clinical trials ongoing in lung cancer

Molecular mechanism analysis revealed the targetable driver mutations in metastatic NSCLC patients. The treatment strategy of patients with metastatic NSCLC underwent significant transformation based on the molecular characteristic. Tyrosine kinase inhibitors (TKI) have provided an illustrative example of the successes in targeting oncogene addiction in lung cancer. Besides these targeted therapies, immune checkpoint inhibitors (ICI) to the treatment of metastatic NSCLC has dramatically changed the prognosis of selected advanced-stage patients. Suitable treatment options also need to explore through clinical trials to confirm.

### EGFR/ALK TKIs

EGFR mutations are observed in 10–20% of patients not of East Asian descent with NSCLC and in approximately 40% of Asian patients.^73^ EGFR gene mutations mostly occur in adenocarcinomas, younger women and girls, and never-smokers,^73,74^ and are rarely identified in cases of lung cancer in smokers, which are usually more malignant than cases of lung cancer in nonsmokers.^75^

The first-generation reversible anti-EGFR tyrosine kinase inhibitors erlotinib and gefitinib were initially approved as second-line therapies in advanced patients after chemotherapy based on the many clinical trials that demonstrated their efficacy in some patients after initial lines of chemotherapy.^76–80^ They are now recommended as first-line therapies in advanced lung cancer patients with EGFR mutations.

Second-generation TKIs are a first-line therapy option for EGFR mutation-positive NSCLC. The LUX Lung 7^81^ study compared afatinib with gefitinib in treatment-naive patients with activating EGFR mutations. The results exhibited improvements in PFS (HR = 0.73, 95% CI: 0.57–0.95, *p* = 0.017); the median PFS values were 11.0 and 10.9 months for the afatinib and gefitinib arms, respectively. However, grade 3–4 EGFR TKI-related toxicities, including skin rash, diarrhea, and stomatitis, were more common with afatinib than with gefitinib. Irreversible second-generation TKIs were developed with the intent to overcome these resistant tumor clones,^82,83^ but the associated clinical trials did not improve overall survival (OS) in T790M-mutant patients.^82^

In squamous cell lung cancer, EGFR mutations are rare. For advanced squamous cell lung cancer, the LUX Lung 8 phase III randomized controlled trial (*N* = 795) reported that afatinib improved PFS (2.6 vs 1.9 months, HR = 0.81, *p* = 0.0103) and OS (7.9 vs 6.8 months, HR = 0.81, *p* = 0.0077) compared with erlotinib as second-line therapy.^84^ The FDA and the European Medicines Agency have approved the use of afatinib in advanced squamous cell lung cancer patients after first-line chemotherapy.

**Fig. 2:**
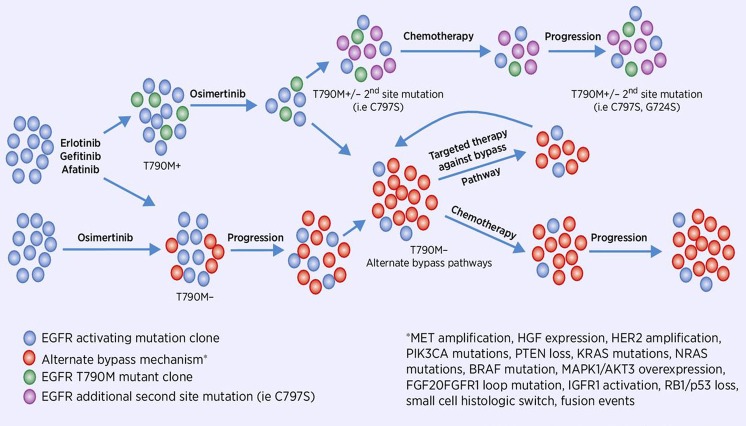
Clonal evaluation of EGFR-mutant lung cancer during therapy. Visual representation of the minimal residual disease and malignant cell progression after anti-EGFR TKIs demonstrating genomic patterns of selection observed in clinical samples during different lines of therapy^164^.

More than 60% of patients develop resistance to first- or second-generation inhibitors. The common mechanism of acquired resistance to EGFR TKIs is the EGFR T790M mutation.^85^ Third-generation inhibitors were created to target the T790M clone while maintaining activity against the original exon19del and L858R mutations (Fig. 2). Osimertinib (AZD9291) is a third-generation tyrosine kinase inhibitor that has received accelerated approval by the FDA based on its targeting of the EGFR T790M mutation. Osimertinib initially gained FDA approval for patients with metastatic EGFR T790M-mutant NSCLC that had progressed on first- or second-generation EGFR TKIs and has subsequently gained approval as a first-line treatment of EGFR-mutant lung cancer. Other third-generation inhibitors are at various stages of development, and have not demonstrated superiority to osimertinib despite antitumor efficacy. Additional EGFR mutations have been detected during therapy. Mechanisms of resistance were also observed in the alternate pathways, such as EGFR amplification, hepatocyte growth factor (HGF) overexpression, FGF2-FGFR1 loop mutations,^86^ MET amplification, PIK3CA mutations (E545K and E542K), PTEN loss, KRAS mutations, NRAS mutations, BRAF mutations, MAPK1/AKT3 overexpression,^87^ HER2 amplification,^88^ IGF1R activation, fusion events,^89^ and RB1/p53 loss, associated with the histologic switch to small-cell cancer.^90^

Anaplastic lymphoma kinase (ALK) gene rearrangements are found in ~5% of patients with NSCLC and generally occur independently of EGFR and KRAS mutations, with a high risk of brain metastases.^91–93^ Three ALK tyrosine kinase inhibitors (TKIs) including crizotinib, ceritinib, and alectinib, have been approved by the FDA for the treatment of ALK-rearranged NSCLC patients.^94^ Crizotinib was the first ALK inhibitor, and acquired resistance to crizotinib is inevitable through several mechanisms.^95–98^ Second- (ceritinib, alectinib, brigatinib) and third-generation (lorlatinib) ALK inhibitors were developed. Alectinib is a second-generation ALK inhibitor and was shown to have high intracranial efficacy treating brain metastases.^99–101^ In ALK-rearranged patients, a variety of ALK resistance mutations can arise after treatment with crizotinib and other ALK inhibitors. To optimize treatment, combinatorial therapies with other targeted agents as well as combinations of ALK inhibitors with immunotherapy warrant further preclinical and clinical investigation.

### Checkpoint inhibitors

Immunotherapy has changed the treatment landscape for cancer patients. Therapies targeting the programmed cell death-1 (PD-1), programmed cell death ligand-1 (PD-L1) and cytotoxic T-lymphocyte-associated antigen-4 (CTLA-4) immune checkpoints have received approval across a wide range of tumor types, including NSCLC. Anti-PD-1/PD-L1 antibodies to treat metastatic NSCLC first entered the clinic in 2015 based on the CheckMate 017 trial.^102^ Then, pembrolizumab and atezolizumab also received regulatory approval in previously treated metastatic NSCLC patients.^103,104^ The results of CheckMate 032 have shown impressive response rates for the nivolumab and nivolumab/ipilimumab arms in relapsed small-cell cancer.^105^ However, immunotherapy is only effective for a small percentage of cancer patients, and the complexity of the tumor immune microenvironment may account for this phenomenon. Alternative immune checkpoints beyond PD-1/PD-L1 and additional therapies must be sought so that more patients can benefit from immune checkpoint therapy.

Several clinic trials using other immune checkpoint inhibitors in lung cancer are ongoing, such as trials of therapies targeting lymphocyte activation gene-3 (LAG-3), T cell immunoglobulin and mucin-containing Protein 3 (TIM-3), V-domain Ig suppressor of T cell activation (VISTA), human endogenous retrovirus‑H long terminal repeat‑associating protein 2 (HHLA2), and T cell Ig and immunoreceptor tyrosine‑based inhibitory motif domain (TIGIT).

### Combination treatment strategies

**Fig. 3:**
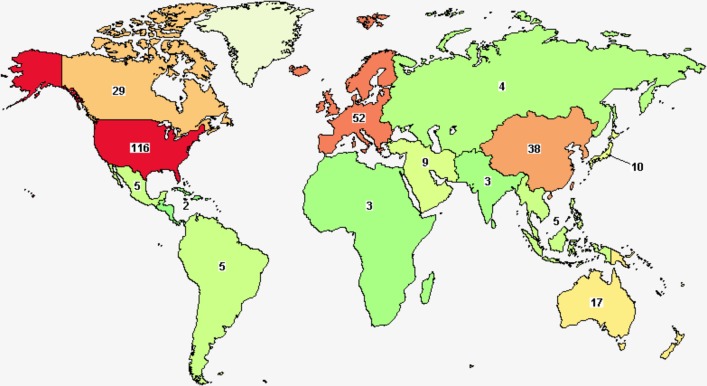
A map of the clinical trials of combination immunotherapy in lung cancer. Combination strategies have shown promise to overcome therapeutic resistance in some clinical trials in lung cancer. Clinical trials studying the combination of immunotherapy with standard therapies (e.g., chemotherapy, radiotherapy, antiangiogenic therapy, and targeted therapy), and combination of checkpoint inhibitors (anti-PD-L1/PD-1 therapies) with other immunotherapy inhibitors are ongoing. In total, 356 studies of combination immunotherapy in lung cancer were found.

As mentioned above, the clinical benefit of ICIs has been proven limited and unsatisfactory, with the overall response rate (ORR) of monotherapy about 10–20%. Current attempts to overcome this limitation include the development of predictive biomarkers and the introduction of combination therapies including ICIs. To improve the efficacy of the checkpoint inhibitor, lots of clinical trials with combination treatment therapy are emerged (Fig. 3).

#### Checkpoint inhibitors combinations with a chemotherapy

For those patients that lack driver mutations and high PD-L1 expression, platinum-based chemotherapy remains the standard of care. The efficacy of chemotherapy is modest. Chemotherapy could induce PD‐L1 expression on tumor cells and infiltrating immune cells.^158^ Immune checkpoint inhibitor therapies, harnessing the immune system, are demonstrating promising results in combination with chemotherapy.^159^

The KEYNOTE-189 trial reported the results of a double-blind phase III study of chemotherapy in combination with pembrolizumab.^160^ This study showed significant improvement in the pembrolizumab arm: a median PFS 8.8 months vs. 4.9 months (HR, 0.52; 95% CI, 0.43–0.64) and an estimated OS of 69.2% vs. 49.4% at 12 months (HR, 0.49; 95% CI, 0.38–0.64). The survival benefit of combination therapy was evident in all subgroups, irrespective of age, sex, performance status, smoking status, brain metastases, PD-L1 expression, and type of platinum therapy used, although toxicity slightly increased. The double-blind, phase III KEYNOTE-407 study exhibited a similar finding in treatment-naive patients with metastatic squamous NSCLC.^106^ The combination of standard chemotherapy with immune checkpoint inhibitors could be a new strategy for first-line therapy in advanced stage NSCLC (Table 1).

**Table 1:** Combinations of checkpoint inhibitors with chemotherapy in clinical trails.

Checkpoint inhibitors	Chemothepapy	Patients enrolled	Clinical setting	Phase	NCT numbers	status	Estimated completion date
Nivolumab	Docetaxel	664	Advanced NSCLC with disease progression on or after platinum-Based Chemotherapy	III	NCT03906071	Recruiting	December 2022
Nivolumab + Ipilimumab	Platinum-based chemotherapy + Nivolumab	112	Chemonaive metastatic or recurrent squamous-cell lung cancer (SqLC)	II	NCT03823625	Recruiting	February 2021
Atezolizumab	Vinorelbine	71	Second-line treatment for patients with stage IV non-small cell lung cancer	II	NCT03801304	Recruiting	July 2022
Trilaciclib + Atezolizumab	Carboplatin + Atezolizumab	105	Untreated extensive stage small cell lung cancer	II	NCT03041311	Active, not recruiting	May 2020
Nivolumab	Tetrahydrouridine	13	Second line therapy for non-small cell lung cancer:PRECISE Trial	II	NCT02664181	Completed	July 2019
M7824 + temozolomide	M7824 + topotecan	57	Relapsed small cell lung cancers	I/II	NCT03554473	Recruiting	January 2024
Nivolumab	carboplatin/paclitaxel or carboplatin/pemetrexed	192	Metastatic or advanced non-small cell lung cancer (NSCLC)	I	NCT02944396	Active, not recruiting	October 2019
Nivolumab	standard chemo-radiotherapy	94	Locally advanced stage iiia/b non-small cell lung carcinoma	II	NCT02434081	Active, not recruiting	August 2020
Nivolumab, Ipilimumab	Carboplatin, Paclitaxel, Pemetrexed, Cisplatin	700	First line therapy in stage IV non-small cell lung cancer	IV	NCT03215706	Recruiting	November 2020

#### Checkpoint inhibitors combinations with radiotherapy

There is evidence that radiotherapy (RT) combined with immunotherapy not only affects the local tumor microenvironment, but also improves systemic disease control.^107^ RT causes the release of tumor-related antigens^163^ and induces a release of some danger signals, such as HMGB1, ATP, and HSP90. There are several clinical trials exploring anti-PD-1/PD-L1 antibodies in combination with radiotherapy in NSCLC patients. In the KEYNOTE‐001 phase I trial, it reported that the PFS with pembrolizumab was significantly longer in patients previously administered RT than in those not treated with RT.^164^As a phase I trial, pembrolizumab was administered at different doses and treatment deliveries. There have been results from phase I, phase II, or phase III (RTOG 3505) and beyond studies, and the results of the RTOG 3505,^165^ which evaluated chemoradiation followed by nivolumab therapy or placebo in locally advanced NSCLC patients in terms of OS, PFS, and toxicity are pending.

#### Checkpoint inhibitor combinations with EGFR TKI strategies

Patients treated with EGFR TKIs eventually develop acquired resistance. It has been reported that activation of the oncogenic EGFR pathway enhances the susceptibility of lung cancers to PD-1 blockade in mouse models, suggesting that combination of PD1 blockade with EGFR TKIs may be a promising therapeutic strategy. As part of the CheckMate 012 study, nivolumab combined with erlotinib exhibited grade 3 toxicities in 19% of patients.^108^ The combination of osimertinib plus durvalumab (phase Ib TATTON trial (NCT02143466)) in pretreated or chemotherapy-naive NSCLC patients showed encouraging clinical activity; however, this combination was associated with a high incidence of diarrhea (53% grade ≥3) in the osimertinib arm and rash (67%) in the combination arm.^109^ Treatment-related grade 3–4 adverse events were observed with the combination therapy. Given the relatively high incidence of treatment-related toxicities associated with the combination of the EGFR TKI and immunotherapy, the combination of the EGFR TKI and immunotherapy should be further investigated.^110^

#### Checkpoint inhibitors (anti-PD-L1/PD-1) combinations with anti-CTLA-4

Strategies combining checkpoint inhibitors with other immune modulatory agents are promising. Combinations with anti-PD-1 or anti-CTLA-4 therapies are being explored in NSCLC as first- and second-line therapies. The CheckMate 012 trial evaluated both the safety and efficacy of nivolumab in combination with ipilimumab as a first-line therapy in advanced NSCLC patients.^111^ In these patients (*n* = 77), grade 3–4 treatment-related AEs occurred in 37% of patients (14 patients) in the group that received ipilimumab every 12 weeks. After a follow-up of 12.8 months, the ORR values were 47 and 38%, respectively. After 2 years of follow-up, the pooled results from both cohorts showed that continued clinical benefit was observed with nivolumab plus ipilimumab in all patients, including those patients with tumor PD-L1 expression ≥1% and ≥50%, regardless of EGFR status, smoking status, or histology. There are several ongoing trials of anti-PD-L1/PD-1 strategies and different combinations in NSCLC (Table 2). These combination therapies are promising.

**Table 2:** combinations of checkpoint inhibitors in clinical trails.

Checkpoint inhibitors	Patients enrolled	Clinical setting	phase	NCT numbers	status	Estimated completion date
Nivolumab + Ipilimumab	1100	Advanced NSCLC	IV	NCT02869789	Recruiting	June 2020
Nivolumab + Ipilimumab	50	Anti-PD-1-axis therapy-resistant advanced non-small cell lung cancer	II	NCT03262779	Recruiting	November 2020
Nivolumab + Ipilimumab + Nintedanib	98	Non small cell lung cancer	I/II	NCT03377023	Recruiting	February 2021
Nivolumab + Ipilimumab	108	After concurrent chemoradiation in patients with unresectable stage III NSCLC	II	NCT03285321	Recruiting	September 2022
Pembrolizumab + Ipilimumab	57	Advanced NSCLC/extensive-disease small cell lung cancer (MK-3475-011/KEYNOTE-011)	I	NCT01840579	Active, not recruiting	June 2020
Nivolumab + Ipilimumab	504	Advanced non-small cell lung cancer (FRACTION-Lung)	II	NCT02750514	Active, not recruiting	April 2021
Nivolumab + Ipilimumab	30	Resectable non-small-cell lung cancer	II	NCT02259621	Recruiting	January 2023
Nivolumab + Ipilimumab	24	Pretreated advanced stage non-small cell lung cancer patients	I	NCT03509584	Not yet recruiting	April 2021

## Treatment advance of targeted therapy in non-small cell lung cancer

Since the targeted therapy was well developed, chemotherapy was no longer the most important treatment for advanced and metastatic NSCLC patients. Meanwhile, chemotherapy cannot differentiate tumor cells and normal cells while working, the treatment-related adverse reactions are dramatically strong therefore being feared by patients. The molecularly targeted therapies with less adverse reactions have prolonged disease control, and ultimately improved long-term survival outcomes in NSCLC patients.

### First-line therapy

#### EGFR-TKIs

EGFR gene sensitivity mutations are the most important discovery in the clinical research of lung cancer in the 21st century. The EGFR gene is one of the most well-studied molecular targets driving lung cancer, and EGFR-targeted therapy has been applied to all stages of advanced NSCLC.

Japanese researchers first used gefitinib in 16 patients with EGFR gene sensitivity mutations, with an effective rate of 75%.^112^ This opened the era of phase III clinical research for targeted therapy in patients with EGFR gene sensitivity mutations. The results of the IPASS, NEJGSG, WJTOG3405, First-SIGNAL, OPTIMAL, EURTAC and LUX-Lung3 trials all showed that patients with EGFR gene sensitivity mutations who were treated with TKIs were significantly better than traditional platinum-containing chemotherapy in the first-line setting.^80,113–117^ For EGFR-mutated NSCLC patients, EGFR-TKIs have a significant advantage in terms of PFS, quality of life and tolerance, thus establishing the role of EGFR-TKIs in first-line setting in advanced EGFR-mutated NSCLC patients.

Osimertinib is a third-generation, irreversible EGFR-TKI that can selectively inhibit both EGFR-TKI-sensitizing and EGFR T790M resistance mutations. The EGFR T790M mutation is the most common mechanism of acquired EGFR resistance; however, the same mutation on both alleles is also present in 1–8% of patients with NSCLC, which may imply a poorer prognosis.^118^ Due to the positive results of the AURA clinical trial, osimertinib has been approved to treat T790M-positive NSCLC patients who have disease progression after EGFR-TKI therapy.^119–121^ In a phase III trial (FLAURA), gefitinib and erlotinib are being compared to osimertinib in previously untreated patients with EGFR-mutant NSCLC. In total, 556 patients were assigned in a 1:1 ratio to receive either osimertinib or a standard EGFR-TKI. In January 2018, it was announced that osimertinib showed superior efficacy over standard EGFR-TKIs, and it also showed a significantly prolonged median progression-free survival compared with standard EGFR-TKIs (18.9 months vs. 10.2 months).^122^ NCCN oncology clinical practice guidelines recommend osimertinib for the first-line treatment of advanced or metastatic EGFR-mutated NSCLC patients (IA).^123^ In April 2018, The US Food and Drug Administration (FDA) approved osimertinib as a first-line treatment for patients with metastatic EGFR-mutant NSCLC.^124^ The FLAURA trial has clearly established osimertinib as a well-tolerated and effective systemic therapy for advanced EGFR mutant NSCLC in the first-line setting. Despite its robust first-line efficacy, further research is needed to determine which sequence of EGFR TKIs will ultimately provide the greatest duration of disease control, the longest overall survival, and the best quality of life. Additionally, as further insights into new treatments for acquired resistance to third-generation EGFR inhibitors emerge, the place of osimertinib in therapeutic strategies may continue to evolve.

EGFR-TKIs enable patients to achieve superior survival, but currently, the efficacy of monotherapy has reached a bottleneck. Targeted therapy combined with anti-angiogenic drugs can improve the tumor microenvironment, which may be a better treatment plan. In a phase II trial (BELIEF), the combination therapy of erlotinib and bevacizumab was evaluated in previously untreated patients. The median progression-free survival was 16 months in the T790M-positive subgroup.^125^ A phase II study (JO25567) was undertaken in Japan to compare erlotinib alone with erlotinib plus bevacizumab, as a first-line therapy in patients with advanced EGFR-mutant NSCLC. This study found that the PFS was longer with the combination treatment than with erlotinib monotherapy (HR = 0.54, *p* = 0.0015).^126^ A subsequent confirmatory phase III study (NEJ 026) was designed to compare erlotinib with erlotinib plus bevacizumab, in patients with untreated EGFR-mutated NSCLC. It showed that the median PFS was 13.3 months compared to 16.9 months (HR = 0.605, *p* = 0.016).^127^ Therefore, bevacizumab plus erlotinib has become a new standard therapy for treatment-naive patients with EGFR-mutated NSCLC.

#### PD-1 targeted immunotherapy as first-line therapy

The proportion of patients who have mutations in driver genes in NSCLC is not high. It accounts for only about 30% of the Western population. The proportion of adenocarcinomas in the Chinese population is higher than that in the Western population, but ~40% of these patients still do not have mutations in driver genes.^128,129^ Platinum-based doublet chemotherapy regimens remain the cornerstone of treatment for patients with recurrent or metastatic NSCLC without EGFR mutations or ALK translocations.^123^

Human immune checkpoint inhibitor (ICI) antibodies can inhibit the PD-1 receptor or PD-L1 and improve antitumor immunity.^130,131^ The PD-1/PD-L1 signaling pathway plays an important role in NSCLC. Lung tumor tissue samples obtained from patients with treatment-naive, advanced NSCLC have demonstrated a high level of PD-L1 expression (TPS ≥ 50%) in ~30% of patients.^132,133^

The Phase III study (KEYNOTE-024) compared pembrolizumab monotherapy with standard platinum-based chemotherapy in adult patients (≥18 years) with previously untreated advanced NSCLC.^134^ 305 patients were randomly assigned to two arms (pembrolizumab, *n* = 154; chemotherapy, *n* = 151). The median OS was 30.0 months with pembrolizumab (95% CI, 18.3 months to not reached) and 14.2 months with chemotherapy (95% CI, 9.8 to 19.0 months). There were less treatment-related grade 3–5 adverse events with pembrolizumab than with chemotherapy (31.2 vs 53.3%). Due to the confirmed favorable benefit-to-risk profile of PD-1 inhibition, the FDA and EMA have approved pembrolizumab monotherapy for the first-line treatment of patients with metastatic NSCLC whose PD-L1 TPS is ≥50%, with no EGFR or ALK mutation.^135^

Immune checkpoint blockades have displayed great potential in cancer therapy and have exhibited a remarkable efficacy compared to conventional treatments for advanced NSCLC. However, not all patients benefit from these agents. Equally important, the development of resistance to anti-PD-1/PD-L1 immunotherapy can lead to the failure and poor prognosis in advanced NSCLC patients. The mechanism underlying the resistance is not fully understood. In addition, little is known about the role of varying levels of PD-L1 positivity, the potential antigen load or mutational load in the tumor, and genetic determinants in the efficacy and resistance associated with anti-PD-1/PD-L1 therapy.

### Subsequent therapy

Because the line of therapy can vary depending on previous treatment with targeted agents, the phrase subsequent therapy has been recently substituted for the terms second-line systemic therapy, third-line systemic therapy, and beyond. Subsequent therapy for patients who have disease progression during or after first-line therapy mostly depend on the specific genetic alteration and the histologic subtype.

Most patients who undergo an initial treatment with first- or second-generation EGFR-TKIs develop resistance and disease progression within a median of 10–12 months.^136^ Hence, third-generation EGFR-TKIs, such as osimertinib and rociletinib, which can selectively target T790M mutants, were developed as second-line treatment options for these patients.^137^ The clinical trials of osimertinib included the AURA phase I, AURA extension phase, AURA2, and AURA3 studies, which supported the efficacy of these agents in T790M-positive advanced NSCLC patients, who had progressed after first-or second-generation EGFR-TKIs.^119,120,138^

Nivolumab is the first immune checkpoint inhibitor (ICI) approved for the second-line treatment of NSCLC. A phase II trial (CheckMate-063) evaluated the efficacy and safety of nivolumab in patients with advanced NSCLC. The ORR was 14.5%, with a median OS of 8.2 months, and a 1-year survival rate of 40.8%.^139^ Another two phase III trials (CheckMate-017 and CheckMate-057) were decisive in recommending immunotherapy for the patients of NSCLC. These studies supported the effectiveness of nivolumab in the second-line setting.^102,140^

One large randomized phase IIb/III trial (KeyNote-010) enrolled 1034 pretreated patients with advanced NSCLC with PD-L1 expression in at least 1% of tumor cells. The OS was significantly prolonged with pembrolizumab versus docetaxel. In a recent update of this trial, the 36-month survival rate was 26.4% (95% CI 14.3–40.1) for untreated patients and 19.0% (15.0–23.4) for previously treated patients.^141,142^ Atezolizumab is a humanized IgG1 monoclonal antibody against PD-L1. The POPLAR study was designed to compare atezolizumab with docetaxel. The results showed the median OS was 12.6 months compared to 9.7 months, respectively (HR 0.73, 95% CI 0.53–0.99; *p* = 0.04). The 3-year OS rate was 18.7% vs 10%, respectively.^143,144^ Additionally, the OAK study was a phase III trial that enrolled 1,225 pretreated NSCLC patients who were randomized to receive atezolizumab or docetaxel. The median OS was 13.8 months vs 9.6 months, respectively (HR 0.73, 95%CI 0.62–0.87; *p* = 0.0003).^104^

### Maintenance therapy

Maintenance therapy is usually used to slowdown the growth of advanced cancer after the initial treatment. It includes two situations: continuation maintenance and switch maintenance. Continuation maintenance chooses one or more drugs which have been used in the first-line therapy. Switch maintenance, also known as early second-line, uses an additional agent after the first-line chemotherapy. Paclitaxel was the first chemotherapeutic agent tested as maintenance therapy.^58^

Approval of erlotinib in the maintenance setting for locally advanced or metastatic NSCLC was based on the results of the randomized, multicenter, placebo-controlled phase III SATURN trial.^145^ In this study, ~2000 patients were randomized to placebo or maintenance erlotinib after platinum-based chemotherapy. After a median follow-up of 11 months, erlotinib showed a statistically significant but very slight advantage in PFS (3.0 vs 2.8 months). In addition, patients with EGFR-mutated adenocarcinoma obtained the greatest outcome benefit.

The INFORM study confirms that, patients receiving EGFR-TKIs as maintenance or subsequent treatment have longer OS than patients who have never received any EGFR TKIs. The positive results from the INFORM trial demonstrate that gefitinib in the maintenance setting leads to significantly improved outcomes for patients with EGFR-mutated advanced NSCLC.^146^

In contrast, a randomized, double-blind, phase III trial (IUNO) demonstrated that, no OS benefit was observed for maintenance erlotinib versus second-line erlotinib treatment in patients with advanced NSCLC without EGFR-activating mutations. Subgroup analyses of OS based on stratification factors, demographics, or baseline characteristics provided results that were consistent with those for the overall population.^147^ based on the lack of benefit observed in this trial, maintenance treatment with erlotinib is no longer considered to be superior in patients with advanced or metastatic NSCLC without EGFR mutation. The maintenance indication is being revised, and maintenance therapy with erlotinib should only be considered for patients with locally advanced or metastatic EGFR-mutated NSCLC.

Using bevacizumab or pemetrexed as continuation maintenance is an option in patients with nonsquamous NSCLC and negative or unknown EGFR mutation status, ROS1 rearrangements, ALK rearrangements, or PD-L1 expression in less than 50%. A randomized study (ECOG 4599) enrolled 878 patients with recurrent or advanced NSCLC and compared chemotherapy with chemotherapy plus bevacizumab.^148^ The median survival was 10.3 vs 12.3 months (hazard ratio for death, 0.79; *p* = 0.003). The median progression-free survival in the two groups was 4.5 vs 6.2 months (hazard ratio for disease progression, 0.66; *P* < 0.001). The POINTBREAK study reported a very slight improvement in PFS (6 vs 5.6 months) when comparing bevacizumab plus pemetrexed versus bevacizumab alone as maintenance therapy.^149^ Similarly, the AVAPERL study using bevacizumab plus pemetrexed versus bevacizumab alone as maintenance therapy, showed a 3.7-month increase in PFS (7.4 vs 3.7 months).^150^ A phase III trial (ATLAS) evaluated bevacizumab with or without erlotinib, after bevacizumab combined with chemotherapy for the first-line treatment of advanced NSCLC.^151^ The results showed that the addition of erlotinib to bevacizumab significantly improved PFS (4.8 vs 3.7 months) but not OS (14.4 vs 13.3 months). The slight impact on survival and increased toxicity of erlotinib combined with bevacizumab indicate that this two-drug maintenance regimen will not be a new standard therapy.

In summary, maintenance therapy is a new treatment option for advanced NSCLC patients with no additional significant side effects beyond those first-line chemotherapy, no significant comorbidities and good PS. The impacts on QoL and economic aspects for patients should be further considered. Predictive factors are needed to select patients who may benefit from maintenance therapy.

### Adjuvant therapy

#### Targeted therapy in early-stage NSCLC

Nearly a third of NSCLC patients have potentially curable early-stage disease. Adjuvant cisplatin-based doublet chemotherapy has been shown to improve overall survival in patients with stage I-III NSCLC.^152^ A meta-analysis named Lung Adjuvant Cisplatin Evaluation (LACE) was conducted to gain a comprehensive understanding of the benefits of adjuvant chemotherapy. The LACE meta-analysis enrolled 4584 patients and had a median follow-up of 5.2 years. The results confirmed a 5.4% overall survival (OS) benefit with adjuvant chemotherapy compared to observation at 5 years (HR = 0.89, 95% CI = 0.82–0.96).^153^ A Cochrane meta-analysis by Burdett et al. also confirmed the benefit of adjuvant chemotherapy in early-stage NSCLC.^154^

To assess the role of targeted agents in adjuvant therapy, several clinical trials are ongoing. The RADIANT study was designed to evaluate the efficacy of erlotinib as an the adjuvant treatment in patients with resected NSCLC.^155^ The results showed that disease-free survival favored erlotinib with an HR of 0.61 (95% CI 0.38–0.91, *p* = 0.039). This difference, however, was not significant according to the statistical design. The results from the RADIANT trial support the need for further adjuvant trials in patients selected by biomarker status. However, a recently presented analysis showed no disease-free survival benefit with erlotinib in patients with EGFR mutations, with an HR of 0.75 (95% CI 0.48–1.16).^156^ In a single-arm phase II trial (SELECT), adjuvant therapy with erlotinib was given for 2 years after surgery to patients with stage IA–IIIA EGFR-mutant NSCLC.^157^ Only two patients recurred during erlotinib therapy among the 24 patients who had disease recurrence. The 2-year disease-free survival was 90%, which was higher than the expected rate of 72%.^158^ The authors commented that the patients who had disease recurrence had a significantly shorter duration of treatment. Therefore, a longer duration of targeted adjuvant treatment may be beneficial for patients after surgery. Since it was a single-arm study, caution needs to be exercised when interpreting the results. Recently, a phase III trial (CTONG1104) was reported. In this study, patients with resected stage II–IIIA EGFR-mutant NSCLC were randomized to either gefitinib for 2 years or vinorelbine plus cisplatin for four cycles.^159^ After a median follow-up of 36.5 months, the gefitinib group had a significantly longer median disease-free survival than vinorelbine plus cisplatin group (28.7 months vs 18 months, HR 0.6, 95% CI 0.42–0.87, *p* = 0.005). Disease-free survival was significantly better with gefitinib at 3 years than with vinorelbine plus cisplatin (34% vs. 27%, *p* = 0.013). Since the data are not yet complete, the OS results have not been reported.

In summary, the available data suggest that adjuvant therapy using EGFR-TKIs could improve PFS, but this advantage has not been translated into prolonged OS. This finding might suggest reason why EGFR-TKIs are unable to eliminate disease with micrometastases.

#### Important ongoing trials

**Fig. 4:**
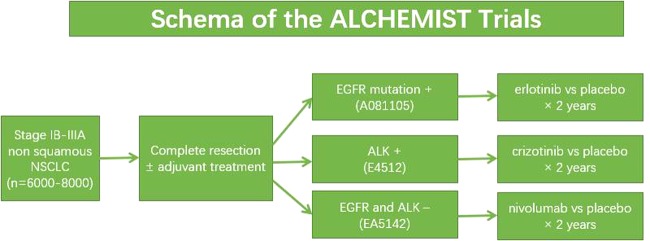
Schematic of the ALCHEMIST trial. 8000 patients are planned to be screened through this trial to facilitate accrual to three substudies: ALCHEMISTEGFR (A081105; NCT02193282), ALCHEMIST-ALK (E4512; NCT02201992), and ALCHEMIST-nivo (EA5142; NCT02595944). In this trial, patients will have first undergone resection and completed standard adjuvant therapy. The patients with EGFR-mutated NSCLC will be randomized 1:1 to either erlotinib or placebo. The patients with ALK-rearranged NSCLC will be randomized 1:1 to crizotinib or placebo. The patients who are negative for EGFR or ALK mutations will be randomized to adjuvant nivolumab or observation for 1 year.

The ALCHEMIST trial, which is also named the Adjuvant Lung Cancer Enrichment Marker Identification and Sequencing Trial, is sponsored by the National Cancer Institute (NCI). To identify and screen patients with EGFR and ALK mutations in early-stage, resected, nonsquamous NSCLC, the study was designed by using an umbrella design (Fig. 4).^160^ Up to August 2018, there had been 309 patients enrolled in the screening trial. The ALCHEMIST trial will answer the question of whether targeted therapy can be part of curative care in NSCLC, and will provide more tumor samples for genomic analysis, which may be an opportunity to advance the understanding of the disease biology.

#### Adjuvant and neo-adjuvant immunotherapy trials

Checkpoint inhibitors targeting the PD1/PD-L1 axis, such as pembrolizumab, nivolumab, and atezolizumab, have completely changed the landscape of treatment in advanced or metastatic lung cancers. Given the promising results seen in the advanced or metastatic diseases settings, these agents are now being assessed as adjuvant treatments in early-stage NSCLC.

The ALCHEMIST-nivo (EA5142; NCT02595944) study is one arm of the ALCHEMIST trial, in this study, patients without EGFR or ALK mutations will be randomized to adjuvant nivolumab versus observation for up to 1 year. A global phase III randomized trial (IMpower010, NCT02486718), is being conducted to evaluate the efficacy and safety of atezolizumab versus best supportive care in patients with resected stage IB–IIIA NSCLC following adjuvant cisplatin-based chemotherapy. Another phase III study (PACIFIC) will compare the PD-L1 inhibitor durvalumab versus placebo in unresectable stage III NSCLC patients following chemoradiation adjuvant therapy.^161^ The interim analysis of the PACIFIC trial has reported a median PFS of 16.8 (durvalumab) vs 5.6 months (placebo), with an HR of 0.52 (95% CI 0.42–0.65, *p* < 0.001). The results of this study provide hope for checkpoint inhibitors as adjuvant therapy in patients with resected NSCLC.

One phase II, open-label, single-arm study (NCT02927301) is designed to confirm the efficacy and safety of atezolizumab as a neo-adjuvant and adjuvant therapy in patients with stage IB–IIIA NSCLC prior to curative-intent resection. Another phase II neo-adjuvant trial (NCT02259621) enrolled patients with stage IB–IIIA NSCLC to evaluate the efficacy of nivolumab before surgery. The initial results presented at ASCO 2017 showed that among the 21 patients who underwent attempted resections, 1 patient was unresectable and 9 patients (43%, 95% CI 24–63%) had a major pathologic response (<10% viable tumor cells in the resection specimen). Recently, the authors have reported that 18 patients (86%) remain alive and recurrence-free, with a median postoperative follow-up of 9 months.^162^ One phase III trial (CheckMate 816, NCT02998528) is designed to compare combination neoadjuvant therapy with nivolumab and ipilimumab or chemotherapy plus nivolumab versus standard neoadjuvant chemotherapy in patients with stage IB–IIIA NSCLC.^163^ These trials will define the role of checkpoint inhibitors as neoadjuvant or adjuvant therapies in resectable NSCLC patients.

## Conclusions and future perspective

In summary, targeted therapies and immunotherapies have changed NSCLC treatment. There have been great advances in lung cancer diagnosis using molecular and immunological methods and theories. In addition to epidermal growth factor receptor (EGFR), new molecular targets are being continuously detected, such as microRNAs, HER3 and immune checkpoint inhibitors, prompting the development of new therapies. Many clinical trials for the agents of targeted therapy and immunotherapy are ongoing and have shown promising and exciting results to date. These trials will help to define the role of targeted therapy in the treatment of lung cancer, including the role of immune monotherapies, combination immunotherapies, and combinations of targeted therapies with immunotherapies, as well as the ideal timing of these therapies and whether they should be used in early-stage versus late-stage disease. Targeted therapy may ultimately change the treatment paradigm for lung cancer, providing hope for patients with limited treatment options. The search of predictive factors of response to targeted drugs remains an important issue of clinical research. Future combination therapies (either targeted therapies or immunotherapies) and better understanding of molecular biomarkers could lead to the ultimate curative option.

## References

[1] Siegel RL, Miller KD, Jemal A (2017). Cancer Statistics, 2017. CA Cancer J. Clin..

[2] Torre LA (2015). Global cancer statistics, 2012. CA Cancer J. Clin..

[3] Ferlay J (2015). Cancer incidence and mortality worldwide: sources, methods and major patterns in GLOBOCAN 2012. Int. J. Cancer.

[4] Halliday PR, Blakely CM, Bivona TG (2019). Emerging targeted therapies for the treatment of non-small cell lung cancer. Curr. Oncol. Rep..

[5] Politi K, Herbst RS (2015). Lung cancer in the era of precision medicine. Clin. Cancer Res..

[6] Herbst RS, Morgensztern D, Boshoff C (2018). The biology and management of non-small cell lung cancer. Nature.

[7] Singh, S. S., Dahal, A., Shrestha, L. & Jois, S. D. Genotype driven therapy for non-small cell lung cancer: resistance, pan inhibitors and immunotherapy. *Curr. Med. Chem.* http://www.eurekaselect.com/170183/article (2019). 10.2174/0929867326666190222183219 30854949

[8] Hay N, Sonenberg N (2004). Upstream and downstream of mTOR. Genes Dev..

[9] Beevers CS, Li F, Liu L, Huang S (2006). Curcumin inhibits the mammalian target of rapamycin-mediated signaling pathways in cancer cells. Int. J. Cancer.

[10] Liu XL (2017). ING5 knockdown enhances migration and invasion of lung cancer cells by inducing EMT via EGFR/PI3K/Akt and IL-6/STAT3 signaling pathways. Oncotarget.

[11] Kawano O (2006). PIK3CA mutation status in Japanese lung cancer patients. Lung Cancer.

[12] Song Z, Yu X, Zhang Y (2016). Mutation and prognostic analyses of PIK3CA in patients with completely resected lung adenocarcinoma. Cancer Med..

[13] Brognard J, Clark AS, Ni Y, Dennis PA (2001). Akt/protein kinase B is constitutively active in non-small cell lung cancer cells and promotes cellular survival and resistance to chemotherapy and radiation. Cancer Res..

[14] Hidalgo M (2006). A phase I and pharmacokinetic study of temsirolimus (CCI-779) administered intravenously daily for 5 days every 2 weeks to patients with advanced cancer. Clin. Cancer Res..

[15] Li C, Lyu J, Meng QH (2017). MiR-93 promotes tumorigenesis and metastasis of non-small cell lung cancer cells by activating the PI3K/Akt pathway via Inhibition of LKB1/PTEN/CDKN1A. J. Cancer.

[16] Ma HP (2019). miRNA-223 is an anticancer gene in human non-small cell lung cancer through the PI3K/AKT pathway by targeting EGFR. Oncol. Rep..

[17] Fumarola C, Bonelli MA, Petronini PG, Alfieri RR (2014). Targeting PI3K/AKT/mTOR pathway in non small cell lung cancer. Biochem Pharmacol..

[18] Fresno Vara JA (2004). PI3K/Akt signalling pathway and cancer. Cancer Treat. Rev..

[19] Perez-Ramirez C (2015). PTEN and PI3K/AKT in non-small-cell lung cancer. Pharmacogenomics.

[20] Wang C, Wang S, Ma F, Zhang W (2018). miRNA328 overexpression confers cisplatin resistance in nonsmall cell lung cancer via targeting of PTEN. Mol. Med. Rep..

[21] Shen H (2014). Alteration in Mir-21/PTEN expression modulates gefitinib resistance in non-small cell lung cancer. PloS ONE.

[22] Han Z (2017). Inhibition of miR-23a increases the sensitivity of lung cancer stem cells to erlotinib through PTEN/PI3K/Akt pathway. Oncol. Rep..

[23] Garofalo M (2011). EGFR and MET receptor tyrosine kinase–altered microRNA expression induces tumorigenesis and gefitinib resistance in lung cancers. Nat. Med..

[24] Zhao J (2017). Synergy between next generation EGFR tyrosine kinase inhibitors and miR-34a in the inhibition of non-small cell lung cancer. Lung cancer.

[25] Zhong M, Ma X, Sun C, Chen L (2010). MicroRNAs reduce tumor growth and contribute to enhance cytotoxicity induced by gefitinib in non-small cell lung cancer. Chem.-Biol. Interact..

[26] Zhou JY (2014). MicroRNA-34a overcomes HGF-mediated gefitinib resistance in EGFR mutant lung cancer cells partly by targeting MET. Cancer Lett..

[27] Downward J (2003). Targeting RAS signalling pathways in cancer therapy. Nat. Rev. Cancer.

[28] Ricciuti B (2016). Targeting the KRAS variant for treatment of non-small cell lung cancer: potential therapeutic applications. Expert Rev. Respir. Med..

[29] Massarelli E (2007). KRAS mutation is an important predictor of resistance to therapy with epidermal growth factor receptor tyrosine kinase inhibitors in non-small-cell lung cancer. Clin. Cancer Res..

[30] Wilhelm SM (2004). BAY 43-9006 exhibits broad spectrum oral antitumor activity and targets the RAF/MEK/ERK pathway and receptor tyrosine kinases involved in tumor progression and angiogenesis. Cancer Res..

[31] Blumenschein GR (2013). Comprehensive biomarker analysis and final efficacy results of sorafenib in the BATTLE trial. Clin. Cancer Res..

[32] Papadimitrakopoulou V (2016). The BATTLE-2 Study: A biomarker-integrated targeted therapy study in previously treated patients with advanced non-small-cell lung cancer. J. Clin. Oncol..

[33] Ferrer I (2018). KRAS-Mutant non-small cell lung cancer: From biology to therapy. Lung Cancer.

[34] Blumenschein GR (2015). A randomized phase II study of the MEK1/MEK2 inhibitor trametinib (GSK1120212) compared with docetaxel in KRAS-mutant advanced non-small-cell lung cancer (NSCLC)dagger. Ann. Oncol..

[35] Gu L, Deng ZJ, Roy S, Hammond PT (2017). A Combination RNAi-chemotherapy layer-by-layer nanoparticle for systemic targeting of KRAS/P53 with cisplatin to treat non-small cell lung cancer. Clin. Cancer Res..

[36] Sun W (2018). miR-202 enhances the anti-tumor effect of cisplatin on non-small cell lung cancer by targeting the Ras/MAPK pathway. Cell. Physiol. Biochem..

[37] Xie Qiong, Yu Zipu, Lu Yuan, Fan Jingya, Ni Yiming, Ma Liang (2018). microRNA‐148a‐3p inhibited the proliferation and epithelial–mesenchymal transition progression of non‐small‐cell lung cancer via modulating Ras/MAPK/Erk signaling. Journal of Cellular Physiology.

[38] MiR-193a-3p is an Important Tumour Suppressor in Lung Cancer and Directly Targets KRAS.pdf. 10.1159/000485491 29183007

[39] The role of STATs in transcriptional control and their impact on cellular function..pdf. 10.1038/sj.onc.1203476 10851045

[40] Lai SY, Johnson FM (2010). Defining the role of the JAK-STAT pathway in head and neck and thoracic malignancies: implications for future therapeutic approaches. Drug Resist. Updat..

[41] Kluge A (2011). Protein inhibitor of activated STAT3 expression in lung cancer. Mol. Oncol..

[42] Ono N (2013). Enhanced antitumor activity of erlotinib in combination with the Hsp90 inhibitor CH5164840 against non-small-cell lung cancer. Cancer Sci..

[43] Chen M, Shao W, He J, Wang D (2010). Role of pemetrexed and platinums combination in patients with non-small cell lung cancer. Curr. Drug Targets.

[44] Hu Y (2014). Inhibition of the JAK/STAT pathway with ruxolitinib overcomes cisplatin resistance in non-small-cell lung cancer NSCLC. Apoptosis.

[45] Hu Y (2016). Enhanced antitumor activity of cetuximab in combination with the Jak inhibitor CYT387 against non-small-cell lung cancer with various genotypes. Mol. Pharm..

[46] Wang N, Zhang T (2018). Downregulation of microRNA-135 promotes sensitivity of non-small cell lung cancer to gefitinib by targeting TRIM16. Oncol. Res..

[47] Patel Manish R., Dash Alexander, Jacobson Blake A., Ji Yan, Baumann Daniel, Ismail Kareem, Kratzke Robert A. (2019). JAK/STAT inhibition with ruxolitinib enhances oncolytic virotherapy in non-small cell lung cancer models. Cancer Gene Therapy.

[48] Boudeau J, Miranda-Saavedra D, Barton GJ, Alessi DR (2006). Emerging roles of pseudokinases. Trends Cell Biol..

[49] Ogiso H (2002). Crystal structure of the complex of human epidermal growth factor and receptor extracellular domains. Cell..

[50] Ferguson KM (2003). EGF activates its receptor by removing interactions that autoinhibit ectodomain dimerization. Mol. Cell..

[51] Burgess AW (2003). An open-and-shut case? Recent insights into the activation of EGF/ErbB receptors. Mol. Cell..

[52] Shi F (2010). ErbB3/HER3 intracellular domain is competent to bind ATP and catalyze autophosphorylation. Proc. Natl Acad. Sci. USA.

[53] Engelman JA (2005). ErbB-3 mediates phosphoinositide 3-kinase activity in gefitinib-sensitive non-small cell lung cancer cell lines. Proc. Natl Acad. Sci. USA.

[54] Yonesaka K (2016). Anti-HER3 monoclonal antibody patritumab sensitizes refractory non-small cell lung cancer to the epidermal growth factor receptor inhibitor erlotinib. Oncogene.

[55] Engelman JA (2007). MET amplification leads to gefitinib resistance in lung cancer by activating ERBB3 signaling. Science.

[56] Huang S (2013). Dual targeting of EGFR and HER3 with MEHD7945A overcomes acquired resistance to EGFR inhibitors and radiation. Cancer Res..

[57] Amin DN, Campbell MR, Moasser MM (2010). The role of HER3, the unpretentious member of the HER family, in cancer biology and cancer therapeutics. Semin. Cell Dev. Biol..

[58] Belani CP (2003). Multicenter, randomized trial for stage IIIB or IV non-small-cell lung cancer using weekly paclitaxel and carboplatin followed by maintenance weekly paclitaxel or observation. J. Clin. Oncol..

[59] Sergina NV (2007). Escape from HER-family tyrosine kinase inhibitor therapy by the kinase-inactive HER3. Nature.

[60] Kawakami H, Yonesaka K (2016). HER3 and its ligand, heregulin, as targets for cancer therapy. Recent Pat. Anticancer Drug Disco..

[61] Malm M, Frejd FY, Ståhl S, Löfblom J (2016). Targeting HER3 using mono- and bispecific antibodies or alternative scaffolds. MAbs.

[62] Meulendijks D (2016). First-in-human phase i study of lumretuzumab, a glycoengineered humanized anti-HER3 monoclonal antibody, in patients with metastatic or advanced HER3-positive solid tumors. Clin. Cancer Res..

[63] Shimizu T (2017). Phase 1 study of new formulation of patritumab (U3-1287) Process 2, a fully human anti-HER3 monoclonal antibody in combination with erlotinib in Japanese patients with advanced non-small cell lung cancer. Cancer Chemother. Pharmacol..

[64] Abramson VG (2017). Phase Ib study of safety and pharmacokinetics of the PI3K Inhibitor SAR245408 with the HER3-neutralizing human antibody SAR256212 in patients with solid tumors. Clin. Cancer Res..

[65] Reynolds KL (2017). A phase I open-label dose-escalation study of the anti-HER3 monoclonal antibody LJM716 in patients with advanced squamous cell carcinoma of the esophagus or head and neck and HER2-overexpressing breast or gastric cancer. BMC Cancer.

[66] Meulendijks D (2017). Phase Ib study of lumretuzumab plus cetuximab or erlotinib in solid tumor patients and evaluation of HER3 and Heregulin as potential biomarkers of clinical activity. Clin. Cancer Res..

[67] Jacob W, James I, Hasmann M, Weisser M (2018). Clinical development of HER3-targeting monoclonal antibodies: Perils and progress. Cancer Treat. Rev..

[68] Nishio M (2015). Phase I study of the HER3-targeted antibody patritumab (U3-1287) combined with erlotinib in Japanese patients with non-small cell lung cancer. Lung Cancer.

[69] Sankyo, D. NCT03260491 (ClinicalTrials.gov, 2018).

[70] Sankyo, D. (ClinicalTrials.gov, 2018).

[71] Yonesaka K (2019). An HER3-targeting antibody-drug conjugate incorporating a DNA topoisomerase I inhibitor U3-1402 conquers EGFR tyrosine kinase inhibitor-resistant NSCLC. Oncogene.

[72] Janne PA (2019). Safety and preliminary antitumor activity of U3-1402: A HER3-targeted antibody drug conjugate in EGFR TKI-resistant, EGFRmNSCLC. J Clin Oncol.

[73] Shigematsu H (2005). Clinical and biological features associated with epidermal growth factor receptor gene mutations in lung cancers. J. Natl. Cancer Inst..

[74] Tsao AS (2016). Scientific advances in lung cancer 2015. J. Thorac. Oncol..

[75] Shih JY (2006). Epidermal growth factor receptor mutations in needle biopsy/aspiration samples predict response to gefitinib therapy and survival of patients with advanced nonsmall cell lung cancer. Int J. Cancer.

[76] Lynch TJ (2004). Activating mutations in the epidermal growth factor receptor underlying responsiveness of non-small-cell lung cancer to gefitinib. N. Engl. J. Med..

[77] Paez JG (2004). EGFR mutations in lung cancer: correlation with clinical response to gefitinib therapy. Science.

[78] Pao W (2004). EGF receptor gene mutations are common in lung cancers from "never smokers" and are associated with sensitivity of tumors to gefitinib and erlotinib. Proc. Natl Acad. Sci. USA.

[79] Mok TS (2009). Gefitinib or carboplatin-paclitaxel in pulmonary adenocarcinoma. N. Engl. J. Med..

[80] Rosell R (2012). Erlotinib versus standard chemotherapy as first-line treatment for European patients with advanced EGFR mutation-positive non-small-cell lung cancer (EURTAC): a multicentre, open-label, randomised phase 3 trial. Lancet Oncol..

[81] Paz-Ares L (2017). Afatinib versus gefitinib in patients with EGFR mutation-positive advanced non-small-cell lung cancer: overall survival data from the phase IIb LUX-Lung 7 trial. Ann. Oncol..

[82] Miller VA (2012). Afatinib versus placebo for patients with advanced, metastatic non-small-cell lung cancer after failure of erlotinib, gefitinib, or both, and one or two lines of chemotherapy (LUX-Lung 1): a phase 2b/3 randomised trial. Lancet Oncol..

[83] Reckamp KL (2014). A phase 2 trial of dacomitinib (PF-00299804), an oral, irreversible pan-HER (human epidermal growth factor receptor) inhibitor, in patients with advanced non-small cell lung cancer after failure of prior chemotherapy and erlotinib. Cancer.

[84] Soria JC (2015). Afatinib versus erlotinib as second-line treatment of patients with advanced squamous cell carcinoma of the lung (LUX-Lung 8): an open-label randomised controlled phase 3 trial. Lancet Oncol..

[85] Yu HA (2013). Analysis of tumor specimens at the time of acquired resistance to EGFR-TKI therapy in 155 patients with EGFR-mutant lung cancers. Clin. Cancer Res..

[86] Terai H (2013). Activation of the FGF2-FGFR1 autocrine pathway: a novel mechanism of acquired resistance to gefitinib in NSCLC. Mol. Cancer Res..

[87] Ercan D (2012). Reactivation of ERK signaling causes resistance to EGFR kinase inhibitors. Cancer Disco..

[88] Takezawa K (2012). HER2 amplification: a potential mechanism of acquired resistance to EGFR inhibition in EGFR-mutant lung cancers that lack the second-site EGFRT790M mutation. Cancer Disco..

[89] Park JH (2016). Activation of the IGF1R pathway potentially mediates acquired resistance to mutant-selective 3rd-generation EGF receptor tyrosine kinase inhibitors in advanced non-small cell lung cancer. Oncotarget.

[90] Kim WJ (2015). Histological transformation from non-small cell to small cell lung carcinoma after treatment with epidermal growth factor receptor-tyrosine kinase inhibitor. Thorac. Cancer.

[91] Shaw AT (2009). Clinical features and outcome of patients with non-small-cell lung cancer who harbor EML4-ALK. J. Clin. Oncol..

[92] Sasaki T, Rodig SJ, Chirieac LR, Janne PA (2010). The biology and treatment of EML4-ALK non-small cell lung cancer. Eur. J. Cancer.

[93] Lazzari C (2014). Targeting ALK in patients with advanced non small cell lung cancer: biology, diagnostic and therapeutic options. Crit. Rev. Oncol. Hematol..

[94] Kwak EL (2010). Anaplastic lymphoma kinase inhibition in non-small-cell lung cancer. N. Engl. J. Med.

[95] Zhang Y, Yin J, Peng F (2017). Acquired resistance to crizotinib in advanced lung adenocarcinoma with MET exon 14 skipping. Lung Cancer.

[96] Heist RS (2016). Acquired resistance to crizotinib in NSCLC with MET Exon 14 Skipping. J. Thorac. Oncol..

[97] Kim S (2013). Heterogeneity of genetic changes associated with acquired crizotinib resistance in ALK-rearranged lung cancer. J. Thorac. Oncol..

[98] Gerlinger M, Norton L, Swanton C (2013). Acquired resistance to crizotinib from a mutation in CD74-ROS1. N. Engl. J. Med..

[99] Tomasini P (2019). Alectinib in the treatment of ALK-positive metastatic non-small cell lung cancer: clinical trial evidence and experience with a focus on brain metastases. Ther. Adv. Respir. Dis..

[100] Lockney NA, Wu AJ (2017). Alectinib for the management of ALK-positive non-small cell lung cancer brain metastases. J. Thorac. Dis..

[101] Gadgeel SM (2014). Safety and activity of alectinib against systemic disease and brain metastases in patients with crizotinib-resistant ALK-rearranged non-small-cell lung cancer (AF-002JG): results from the dose-finding portion of a phase 1/2 study. Lancet Oncol..

[102] Brahmer J (2015). Nivolumab versus Docetaxel in Advanced Squamous-Cell Non-Small-Cell Lung Cancer. N. Engl. J. Med..

[103] Herbst RS (2016). Pembrolizumab versus docetaxel for previously treated, PD-L1-positive, advanced non-small-cell lung cancer (KEYNOTE-010): a randomised controlled trial. Lancet.

[104] Rittmeyer A (2017). Atezolizumab versus docetaxel in patients with previously treated non-small-cell lung cancer (OAK): a phase 3, open-label, multicentre randomised controlled trial. Lancet.

[105] Antonia SJ (2016). Nivolumab alone and nivolumab plus ipilimumab in recurrent small-cell lung cancer (CheckMate 032): a multicentre, open-label, phase 1/2 trial. Lancet Oncol..

[106] Paz-Ares L (2018). Pembrolizumab plus chemotherapy for squamous non-small-cell lung cancer. N. Engl. J. Med..

[107] Ko EC, Raben D, Formenti SC (2018). The Integration of radiotherapy with immunotherapy for the treatment of non-small cell lung cancer. Clin. Cancer Res..

[108] Gettinger S (2018). Nivolumab plus erlotinib in patients with EGFR-mutant advanced NSCLC. J. Thorac. Oncol..

[109] Chih-Hsin Yang J (2019). Osimertinib plus durvalumab versus osimertinib monotherapy in EGFR T790M-positive NSCLC following previous EGFR TKI therapy: CAURAL brief report. J. Thorac. Oncol..

[110] Ahn MJ (2017). EGFR TKI combination with immunotherapy in non-small cell lung cancer. Expert Opin. Drug Saf..

[111] Hellmann MD (2017). Nivolumab plus ipilimumab as first-line treatment for advanced non-small-cell lung cancer (CheckMate 012): results of an open-label, phase 1, multicohort study. Lancet Oncol..

[112] Asahina H (2006). A phase II trial of gefitinib as first-line therapy for advanced non-small cell lung cancer with epidermal growth factor receptor mutations. Br. J. Cancer.

[113] Maemondo M (2010). Gefitinib or chemotherapy for non-small-cell lung cancer with mutated EGFR. N. Engl. J. Med..

[114] Mitsudomi T, Yatabe Y (2010). Epidermal growth factor receptor in relation to tumor development: EGFR gene and cancer. FEBS J..

[115] Han JY (2012). First-SIGNAL: first-line single-agent iressa versus gemcitabine and cisplatin trial in never-smokers with adenocarcinoma of the lung. J. Clin. Oncol..

[116] Zhou C (2011). Erlotinib versus chemotherapy as first-line treatment for patients with advanced EGFR mutation-positive non-small-cell lung cancer (OPTIMAL, CTONG-0802): a multicentre, open-label, randomised, phase 3 study. Lancet Oncol..

[117] Yang JC (2013). Symptom control and quality of life in LUX-Lung 3: a phase III study of afatinib or cisplatin/pemetrexed in patients with advanced lung adenocarcinoma with EGFR mutations. J. Clin. Oncol..

[118] Cross DA (2014). AZD9291, an irreversible EGFR TKI, overcomes T790M-mediated resistance to EGFR inhibitors in lung cancer. Cancer Discov..

[119] Goss G (2018). CNS response to osimertinib in patients with T790M-positive advanced NSCLC: pooled data from two phase II trials. Ann. Oncol..

[120] Goss G (2016). Osimertinib for pretreated EGFR Thr790Met-positive advanced non-small-cell lung cancer (AURA2): a multicentre, open-label, single-arm, phase 2 study. Lancet Oncol..

[121] Yang JC (2017). Osimertinib in pretreated T790M-positive advanced non-small-cell lung cancer: AURA study phase II extension component. J. Clin. Oncol..

[122] Soria JC (2018). Osimertinib in untreated EGFR-mutated advanced non-small-cell lung cancer. N. Engl. J. Med..

[123] Ettinger DS (2018). NCCN guidelines insights: non-small cell lung cancer, version 5.2018. J. Natl. Compr. Cancer Netw..

[124] FDA Approves Frontline Osimertinib for NSCLC.onclive.com.

[125] Rosell R (2017). Erlotinib and bevacizumab in patients with advanced non-small-cell lung cancer and activating EGFR mutations (BELIEF): an international, multicentre, single-arm, phase 2 trial. Lancet Respir Med..

[126] Seto T (2014). Erlotinib alone or with bevacizumab as first-line therapy in patients with advanced non-squamous non-small-cell lung cancer harbouring EGFR mutations (JO25567): an open-label, randomised, multicentre, phase 2 study. Lancet Oncol..

[127] Furuya N (2018). Phase III study comparing bevacizumab plus erlotinib to erlotinib in patients with untreated NSCLC harboring activating EGFR mutations: NEJ026. J. Clin. Oncol..

[128] Li T, Kung HJ, Mack PC, Gandara DR (2013). Genotyping and genomic profiling of non-small-cell lung cancer: implications for current and future therapies. J. Clin. Oncol..

[129] Shi Y (2014). A prospective, molecular epidemiology study of EGFR mutations in Asian patients with advanced non-small-cell lung cancer of adenocarcinoma histology (PIONEER). J. Thorac. Oncol..

[130] Ma W, Gilligan BM, Yuan J, Li T (2016). Current status and perspectives in translational biomarker research for PD-1/PD-L1 immune checkpoint blockade therapy. J. Hematol. Oncol..

[131] Kerr KM, Nicolson MC (2016). Non-Small Cell Lung Cancer, PD-L1, and the Pathologist. Arch. Pathol. Lab. Med..

[132] Reck M (2016). Pembrolizumab versus Chemotherapy for PD-L1-Positive Non-Small-Cell Lung Cancer. N. Engl. J. Med..

[133] Langer CJ (2016). Carboplatin and pemetrexed with or without pembrolizumab for advanced, non-squamous non-small-cell lung cancer: a randomised, phase 2 cohort of the open-label KEYNOTE-021 study. Lancet Oncol..

[134] Reck M (2019). Updated analysis of KEYNOTE-024: pembrolizumab versus platinum-based chemotherapy for advanced non-small-cell lung cancer with PD-L1 tumor proportion score of 50% or greater. J. Clin. Oncol..

[135] KEYTRUDAR? (pembrolizumab). Full Prescribing Information (Merck & Co., Inc., Whitehouse Station, NJ, USA 2017).

[136] Morgillo F, Della Corte CM, Fasano M, Ciardiello F (2016). Mechanisms of resistance to EGFR-targeted drugs: lung cancer. ESMO open..

[137] Singh M, Jadhav HR (2018). Targeting non-small cell lung cancer with small-molecule EGFR tyrosine kinase inhibitors. Drug Discov. Today.

[138] Mok TS (2017). Osimertinib or platinum-pemetrexed in EGFR T790M-positive lung cancer. N. Engl. J. Med..

[139] Gettinger S (2018). Five-year follow-up of nivolumab in previously treated advanced non-small-cell lung cancer: results from the CA209-003 study. J. Clin. Oncol..

[140] Vokes EE (2018). Nivolumab versus docetaxel in previously treated advanced non-small-cell lung cancer (CheckMate 017 and CheckMate 057): 3-year update and outcomes in patients with liver metastases. Ann. Oncol..

[141] Garon EB (2015). Pembrolizumab for the treatment of non-small-cell lung cancer. N. Engl. J. Med..

[142] Leighl NB (2019). Pembrolizumab in patients with advanced non-small-cell lung cancer (KEYNOTE-001): 3-year results from an open-label, phase 1 study. Lancet Respiratory Med..

[143] Fehrenbacher L (2016). Atezolizumab versus docetaxel for patients with previously treated non-small-cell lung cancer (POPLAR): a multicentre, open-label, phase 2 randomised controlled trial. Lancet.

[144] Mazières J (2018). 136PD_PR 3-year survival and duration of response in randomized phase II study of atezolizumab (atezo) vs docetaxel (doc) in 2L+ NSCLC (POPLAR). J. Thorac. Oncol..

[145] Cappuzzo F (2010). Erlotinib as maintenance treatment in advanced non-small-cell lung cancer: a multicentre, randomised, placebo-controlled phase 3 study. Lancet Oncol..

[146] Zhao H (2015). Final overall survival results from a phase III, randomized, placebo-controlled, parallel-group study of gefitinib versus placebo as maintenance therapy in patients with locally advanced or metastatic non-small-cell lung cancer (INFORM; C-TONG 0804). J. Thorac. Oncol..

[147] Cicènas Saulius, Geater Sarayut Lucien, Petrov Petar, Hotko Yevgeniy, Hooper Gregory, Xia Fan, Mudie Nadejda, Wu Yi-Long (2016). Maintenance erlotinib versus erlotinib at disease progression in patients with advanced non-small-cell lung cancer who have not progressed following platinum-based chemotherapy (IUNO study). Lung Cancer.

[148] Sandler A (2006). Paclitaxel-carboplatin alone or with bevacizumab for non-small-cell lung cancer. N. Engl. J. Med..

[149] Patel JD (2013). PointBreak: a randomized phase III study of pemetrexed plus carboplatin and bevacizumab followed by maintenance pemetrexed and bevacizumab versus paclitaxel plus carboplatin and bevacizumab followed by maintenance bevacizumab in patients with stage IIIB or IV nonsquamous non-small-cell lung cancer. J. Clin. Oncol..

[150] Barlesi F (2013). Randomized phase III trial of maintenance bevacizumab with or without pemetrexed after first-line induction with bevacizumab, cisplatin, and pemetrexed in advanced nonsquamous non-small-cell lung cancer: AVAPERL (MO22089). J. Clin. Oncol..

[151] Johnson BE (2013). ATLAS: randomized, double-blind, placebo-controlled, phase IIIB trial comparing bevacizumab therapy with or without erlotinib, after completion of chemotherapy, with bevacizumab for first-line treatment of advanced non-small-cell lung cancer. J. Clin. Oncol..

[152] Chemotherapy in non-small cell lung cancer: a meta-analysis using updated data on individual patients from 52 randomised clinical trials. (1995). Non-small Cell Lung Cancer Collaborative Group. BMJ.

[153] Pignon JP (2008). Lung adjuvant cisplatin evaluation: a pooled analysis by the LACE Collaborative Group. J. Clin. Oncol..

[154] Burdett, S. et al. Adjuvant chemotherapy for resected early-stage non-small cell lung cancer. *Cochrane Database Syst Rev.* CD011430, (2015). 10.1002/14651858.CD011430 PMC10542092 25730344

[155] Kelly K (2015). Adjuvant erlotinib versus placebo in patients with stage IB-IIIA non-small-cell lung cancer (RADIANT): a randomized, double-blind, phase III trial. J. Clin. Oncol..

[156] Shepherd Fa,EWEEANK (2015). Common and rare EGFR mutations (EGFRm) in the RADIANT trial: final follow-up with 5 year data. J. Clin. Oncol..

[157] Pennell Na,NJWCJE (2014). SELECT: a multicenter phase II trial of adjuvant erlotinib in resected early-stage EGFR mutation-positive NSCLC. J. Clin. Oncol..

[158] Janjigian YY (2011). Impact on disease-free survival of adjuvant erlotinib or gefitinib in patients with resected lung adenocarcinomas that harbor EGFR mutations. J. Thorac. Oncol..

[159] Zhong WZ (2018). Gefitinib versus vinorelbine plus cisplatin as adjuvant treatment for stage II-IIIA (N1-N2) EGFR-mutant NSCLC (ADJUVANT/CTONG1104): a randomised, open-label, phase 3 study. Lancet Oncol..

[160] Govindan R (2015). ALCHEMIST trials: a golden opportunity to transform outcomes in early-stage non-small cell lung cancer. Clin. Cancer Res..

[161] Antonia SJ (2017). Durvalumab after chemoradiotherapy in stage III non-small-cell lung cancer. N. Engl. J. Med..

[162] Forde PM (2018). Neoadjuvant PD-1 blockade in resectable lung cancer. N. Engl. J. Med..

[163] Yeh J, Marrone KA, Forde PM (2018). Neoadjuvant and consolidation immuno-oncology therapy in stage III non-small cell lung cancer. J. Thorac. Dis..

[164] Murtuza A (2019). Novel third-generation EGFR tyrosine kinase inhibitors and strategies to overcome therapeutic resistance in lung cancer. Cancer Res..

[165] Gerber David E., Urbanic James J., Langer Corey, Hu Chen, Chang I-Fen, Lu Bo, Movsas Benjamin, Jeraj Robert, Curran Walter J., Bradley Jeffrey D. (2017). Treatment Design and Rationale for a Randomized Trial of Cisplatin and Etoposide Plus Thoracic Radiotherapy Followed by Nivolumab or Placebo for Locally Advanced Non–Small-Cell Lung Cancer (RTOG 3505). Clinical Lung Cancer.

